# Experience versus complication rate in third molar surgery

**DOI:** 10.1186/1746-160X-2-14

**Published:** 2006-05-25

**Authors:** Waseem Jerjes, Mohammed El-Maaytah, Brian Swinson, Bilquis Banu, Tahwinder Upile, Sapna D'Sa, Mohammed Al-Khawalde, Boussad Chaib, Colin Hopper

**Affiliations:** 1 Honorary Lecturer, Department of Oral & Maxillofacial Surgery, Eastman Dental Institute & University College London Hospitals, 256 Gray's Inn Road, London WC1X 8LD, UK; 2 Specialist Registrar, Department of Oral and Maxillofacial Surgery, University College London Hospitals, London, UK; 3 Specialist Registrar, Department of Oral and Maxillofacial Surgery, University College London Hospitals, London, UK; 4 Specialist in Surgical Dentistry, Department of Oral & Maxillofacial Surgery, Eastman Dental Institute for Oral Healthcare Sciences, London, UK; 5 Specialist Registrar, Head & Neck Surgery Unit, University College London Hospitals, London, UK; 6 Specialist Registrar, Department of Oral and Maxillofacial Surgery, University College London Hospitals, London, UK; 7 Specialist, Department of Oral & Maxillofacial Surgery, Royal Medical Services, Amman, Jordan; 8 Reasearcher, Royal Free & University College Medical School, London, UK; 9 Head Of Academic Surgical Unit, Senior Lecturer/Consultant Oral & Maxillofacial Surgeon, Eastman Dental Institute & University College London Hospitals, London, UK

## Abstract

**Objectives:**

The records of 1087 patients who underwent surgical removal of third molar teeth were prospectively examined to analyse the possible relationship between postoperative complications and the surgeon's experience parameter.

**Method and materials:**

Seven surgeons (three specialists in surgical dentistry [specialists SD] and four oral and maxillofacial Senior House Officers [OMFS residents]) carried out the surgical procedures. For each patient, several variables were recorded including age, gender, radiographic position of extracted teeth, treating surgeon, duration of surgery and postoperative complications.

**Results:**

Analysis of the data revealed some differences in the incidence of complications produced by the specialists SD and OMFS residents. The main statistically relevant differences were increase the incidences of trismus, nerve paraesthesia, alveolar osteitis and infection in the resident-treated group, while the specialist-treated group showed higher rates of post-operative bleeding.

**Conclusion:**

The higher rate of postoperative complications in the resident-treated group suggests that at least some of the complications might be related to surgical experience.

Further work needs to compare specialists of training programmes with different years of experience, using large cross – sectional studies.

## Introduction

Dentoalveolar surgery and especially surgical removal of third molar teeth continues to be the most common surgical procedure performed in the speciality of oral & maxillofacial surgery.

The surgical removal of third molar teeth may result in a number of complications including pain, swelling, bleeding, alveolar osteitis (dry socket) or nerve dysfunction [1]. The factors that usually contribute to such problems are numerous and include the patient, tooth-related and the surgeon's operative experience [2].

Although careful attention to surgical details, including proper patient preparation, asepsis, meticulous management of hard and soft tissue, controlled force when applying surgical instruments, haemostasis and adequate post operative instructions may help to reduce this rate of complications it has not been proven to eliminated them. Other parameters found to affect the complication rate include age [3], gender [4], and the surgeon's experience [5,6].

Several authorities have suggested the use of antibiotics placed in the alveolar socket to decrease the bacterial plaque and thereby reduce inflammation, pain and trismus [7,8]. Ragno and Szkutnik [9] recommended the use of chlorhexidine mouth rinses prior to the surgical extraction of impacted third molars to reduce postoperative complications. Penarrocha et al. [10] produced evidence that improving the oral hygiene of the patient preoperatively helps to reduce the rate of postoperative complications, most noticeably in pain.

The literature that compares the experience of surgeons to postoperative complications is sparse as it may have implications in the training of junior surgeons [11].

Sisk et al. [6] investigated the effect of the experience of the surgeon on the complication rate following surgical removal of third molar teeth by comparing an oral surgery faculty group to resident group in the same faculty. They showed that complications were numerous after removal of teeth classified as a partially or completely impacted within bone and also that less experienced surgeons had significantly higher incidences of complications.

Handelman et al. [12] carried out a study to assess the postoperative complications in patients who had undergone surgical removal of third molars by OMFS residents and were compared with those of patients whose extractions were performed by general dentistry residents. They showed that there was no significant difference in complication rates between the two groups, but the pain relief requirements, for patients treated by general dentistry residents, was shown to be higher. This was shown not related to the level of experience but to the type of analgesics administered following the treatment.

Berge and Gilhuus-Moe [13] compared postoperative complications following surgical removal of third molars in two groups of patients. Surgery was performed on the first group by four general dental practitioners and on the second group by a consultant oral surgeon. An increased rate of postoperative alveolar osteitis, pain and increased duration of surgery was found in the general practitioners group.

de Boer et al. [14] showed higher complication rates following third molar surgery in the hands of residents in alveolar osteitis, swelling and post-operative bleeding. Senior staff in the same study showed higher rates of post-operative infection and paraesthesia.

Generally speaking, inexperience of the surgeon has been shown to relate to increased postoperative complications [6,13]. Other studies have, however, failed to reveal any correlation between the experience of the surgeon and postoperative complications [12].

The aim of this study was to compare the incidence of complications following third molar surgery in the hands of a group of surgeons, specialists and residents, to examine whether the experience parameter has a major or minor influence on the results.

## Materials and methods

The records of 1087 patients undergoing surgical removal of third molar teeth were prospectively collected. This included 569 cases in which the removal of third molar teeth in outpatients was performed by three specialists in surgical dentistry in the Department of Oral and Maxillofacial Surgery, Eastman Dental Hospital, London. The prospective records of 518 cases in which the same procedure was performed during the same period by 4 residents (senior house officers) in oral and maxillofacial surgery were also recorded. The cases were analysed to examine incidence of any postoperative complications.

For each patient, the following data was collected:

• Age

• Gender

• Radiographic position (fully erupted, partially impacted or fully impacted)

• Treating surgeon (specialist or resident)

• Surgical technique (described below)

• Closeness to inferior dental nerve (tooth ≤ 2 mm is considered to be close)

• Duration of surgery

• Postoperative complications (measured 1 week postoperatively)

1. Inflammation: local reddening and swelling of the area following the surgical insult.

2. Infection: opening a flap and exposing the underlying tissue to various microbes can lead to infection; patients were recorded as having an infection if they presented with severe pain, swelling and inflammation after the initial swelling subsided.

3. Abscess: abscess can be felt clinically by bi-manual palpation; associated signs and symptoms include pain, pyrexia, swelling, inflammation, trismus and pus discharge.

4. Trismus: our protocol defines trismus as a mouth opening (interincisal distance) of <25 mm postoperatively.

5. Swelling: very difficult to assess especially in a clinical field; in our study we considered soft tissue swelling as an "obvious facial asymmetry".

6. Bleeding: no patient presented with any haemorrhage, any patient who presented with continuous blood loss from the socket is recorded as have postoperative bleeding.

7. Sore throat: was recorded as a complication in patients who presented with pharyngeal pain and dysphagia and reddening of the area on clinical examination.

8. Alveolar osteitis [dry socket]: was recorded as a complication in patients who presented with dull aching pain in an inflamed tooth socket.

9. Delayed healing: a patient was recorded as having delayed clinical healing when further surgical treatment was required on a postoperative visit (e.g. re-suturing).

10. Temporary and permanent nerve dysfunction: nerve paraesthesia data were either related to the inferior alveolar nerve or the lingual nerve. All patients with paraesthesia were followed up for the first four weeks following surgery, six months and up to two years, with any patient beyond this time being considered to have permanent nerve dysfunction.

11. Pain: the most common postoperative complication following third molar surgery; this complication was not investigated in our study.

All surgical procedures were performed in three similar clinics, equipped with similar surgical instruments, rotary and irrigation devices and materials (sutures and haemostatic agents). Prior to surgery, each patient was informed of possible complications including the possible risk of nerve damage during the procedure and provided fully informed consent.

Local anaesthesia was applied (2% Lidocaine with 1:100,000 epinephrine) by local tissue infiltration and inferior alveolar nerve block injection, and no more than five cartridges were given to any single patient.

Surgical approach was implemented in all cases. An envelope mucoperiosteal flap was reflected and bone was removed with a round bur in a straight handpiece. Sectioning of the teeth was carried out using a fissure bur. The sectioning in all cases was performed from the root bifurcation area to the occlusal surface; no other sectioning technique was implemented. Bone removal and sectioning of the tooth was performed under continuous irrigation with sterile saline solution at room temperature. The wound was carefully irrigated and any bony spicules removed. The flap was then repositioned and sutured with 4–0 Vicryl. No lingual flap was employed in any of the cases. No patient in this study underwent coronectomy.

Immediately postoperatively all patients were given written instructions about wound care and possible complications in the post-operative period. For all patients, metronidazole (400 mg three times daily for five days) was prescribed as an antimicrobial agent; it is well documented in the literature that metronidazole is the standard medication used following this kind of surgery as it covers most of the spectrum of the microbial infections. Ibuprofen (400 mg three times daily for five days) was prescribed as an analgesic. All patients in this study were reviewed seven days postoperatively.

The cases were distributed among specialists and residents randomly regardless of patient's age, gender or even complexity of surgery. Patients were required to undergo removal of at least one mandibular third molar tooth for inclusion in this study.

No surgical exploration was implemented for any of the patients presented with paraesthesia for less than two years. Patients who continued to have this symptom over two years (permanent) were reviewed to assess their condition and were advised to undergo surgery to explore the area that could include undertaking microneurosurgical repair for the appropriate cases.

### Statistical methods

The adverse outcomes from surgery were summarised as frequencies separately according to the grade of the surgeon undertaking the procedure. The Chi-squared statistic was used to test for differences in the case-mix between the surgical grades. The odds ratio (and associated 95% confidence interval) for each adverse outcome was calculated to compare the likelihood of a patient suffering that outcome between the surgical grades, such that an odds ratio greater than one indicated greater likelihood of complication in resident-treated group, whereas an odds ratio lower than one indicated the converse.

## Results

The 1087 treated patients had a mean age of 23.3 years and there was a slight female predominance (Table 1). The majority of teeth were partially impacted 857/1087 (78.8%) and around three quarters 843/1087 (77.6%) had roots that appeared radiographically to be within or less than 2 mm from the inferior alveolar nerve. The mean time to complete surgery was 18 minutes. The shortest surgery was completed in 4 minutes and the longest 39 minutes. The specialists treated slightly more patients 569/1087 (52.3%) than the OMFS residents did.

**Table 1:** Profile of treated cases

Category	Description	n (%)
Gender	Male	501 (46.1)
	Female	586 (53.9)
		
Age (years)	Mean	23.3
	Median	22.0
	SD	4.2
	Range	17 – 36
		
Degree of impaction of 3^rd ^molar	Fully erupted	104 (9.6)
	Partially erupted	857 (78.8)
	Fully impacted	126 (11.6)
		
Proximity to inferior alveolar nerve	> 2 mm	244 (22.4)
	<= 2 mm	843 (77.6)
		
Duration of surgery (mins)	Mean	18.1
	Median	18.0
	SD	7.3
	Range	4 – 39
		
Seniority of surgeon	Resident	518 (47.7)
	Specialist	569 (52.3)

The OMFS residents treated more female 303/518 (58.3%) patients than their senior colleagues (Table. 2), while the specialists treated more male patients 286/569 (50.3%). Both of the surgeons treated almost similar number of fully erupted and partially impacted teeth, although, the specialists were noted to have removed more teeth reported as fully impacted 92/569 (16.2%). The residents have treated more patients with wisdom teeth reported to be close to the inferior alveolar nerve 415/518 (80.1%). Age of patients was normally distributed between the two groups.

**Table 2:** Case-mix in relation to seniority of surgeon

Category	Description	Surgeon		X^2 ^(df)	P-value
		Resident n (%)	Specialist n (%)		
Patient gender	Male	215 (41.5)	286 (50.3)		
	Female	303 (58.5)	283 (49.7)	8.37 (1)	0.004
					
Degree of impaction of 3^rd ^molar	Fully erupted	60 (11.6)	44 (7.7)		
	Partially erupted	424 (81.9)	433 (76.1)		
	Fully impacted	34 (6.6)	92 (16.2)	26.92 (2)	<0.001
					
Proximity to inferior alveolar nerve	> 2 mm	103 (19.9)	141 (24.8)		
	<= 2 mm	415 (80.1)	428 (75.2)	3.73 (1)	0.053
					
Patient age group	17–20 years	139 (26.8)	156 (27.4)		
	21–25 years	257 (49.6)	273 (48.0)		
	26–30 years	77 (14.9)	75 (13.2)		
	31+ years	45 (8.7)	65 (11.4)	2.74 (3)	0.434
					
**Total number of cases treated**		**518 (100.0)**	**569 (100.0)**		

Complications in the resident-treated group were slightly higher but statistically insignificant in terms of swelling 62/518 (P = 0.643), sore throat 9/518 (P = 0.117), delayed healing 14/518 (P = 0.129) and abscess formation 7/518 (P = 0.860) (Table. 3). A significant statistical difference in complication rate was noticed in trismus 74/518 (P = 0.003), dry socket 99/518 (P = < 0.001) and post-operative infection 54/518 (P = < 0.001), and this was more noted in the resident-treated group. Post-operative bleeding (33/569) was the only significant complication (P = 0.020) that was reported in the specialist-treated group.

**Table 3:** incidence of each type of post-operative complication in relation to seniority of the surgeon undertaking the procedure.

Complication	Surgeon		OR (95% CI)	P-value
	Resident n (%)	Specialist n (%)	complication in patient treated by Resident	
Trismus	74 (14.3)	49 (8.6)	1.77 (1.21, 2.59)	0.003
Swelling	62 (12.0)	63 (11.1)	1.09 (0.75, 1.59)	0.643
Bleeding	15 (2.9)	33 (5.8)	0.48 (0.26, 0.90)	0.020
Sore throat	9 (1.7)	4 (0.7)	2.50 (0.76, 8.16)	0.117
Dry socket	99 (19.1)	39 (6.9)	3.21 (2.17, 4.75)	<0.001
Delayed healing	14 (2.7)	8 (1.4)	1.95 (0.81, 4.68)	0.129
Abscess	7 (1.4)	7 (1.2)	1.10 (0.38, 3.16)	0.860
Infection	54 (10.4)	25 (4.4)	2.53 (1.55, 4.13)	<0.001
				
Lip numbness at 2 weeks	15 (2.9)	4 (0.7)	4.21 (1.39, 12.77)	0.012
				
Tongue numbness at 2 weeks	24 (4.6)	6 (1.1)	4.56 (1.85, 11.24)	<0.001
Lip numbness at 2 years	7 (1.4)	1 (0.2)	7.78 (0.95, 63.46)	0.056
				
Tongue numbness at 2 years	9 (1.7)	2 (0.4)	5.01 (1.08, 23.31)	0.048
				
**Any complication**	**330 (63.7)**	**223 (39.2)**	**2.72 (2.13, 3.48)**	**<0.001**

The resident-treated group were more likely to develop inferior dental (15/518) (P = 0.012) and lingual nerve paraesthesia (24/518) (P = < 0.001) within the first two weeks following surgery. This group were also more likely to sustain such a complication {(lip numbness P = 0.056), (tongue numbness P = 0.048)} for the first two years following surgery.

The incidence of any complication is significant when the two treated groups are compared; the benefit was for the specialist-treated group 223/569 (39.2%).

## Discussion

In general, we found that the Oral and Maxillofacial residents reported a higher incidence of trismus, nerve paraesthesia, alveolar osteitis and infection, while bleeding was the only parameter that showed a higher incidence in the hands of the specialists. The incidence of any complications reached 63.7% in the resident-treated group and was highly significant (P = < 0.001) when compared to the specialist-treated group. This data was found to be consistent with previous studies^6^.

### Trismus and swelling

Trismus and swelling are subjective findings and difficult to measure objectively, despite being readily observable. Various techniques have been proposed and implemented to measure them [15,16]. In this study, trismus and swelling were recorded as complications regardless of their severity. The incidence of trismus was higher in the hands of the residents, which may be related to the effect of prolonged surgery on the masticatory muscles. This was found to be consistent with some studies [4,6,13] and inconsistent with others [12,14]. There was no difference between the two groups in terms of swelling. These results are in line with de Boer et al. [14].

Previous studies on the reduction of swelling by administration of dexamethasone have demonstrated a marked effect on the speed of recovery of the patient from the procedure [17]. The administration of antimicrobials was not considered to reduce post-operative trismus and swelling since they are the effect of surgical trauma.

Many surgeons feel that there is no necessity to try to reduce postoperative swelling and trismus as it is a prophylactic phenomenon and usually subsides after 3–5 days in any event.

### Wound management

The incidence of bleeding following third molar extractions was twice as high in the specialist-treated group than in the resident-treated group. These findings are not, however, in line with previous publications [1,6,14].

However, when it comes to infection, OMFS residents' patients are twice as likely to develop infection. This could be related to the fact that those surgeons treated more female patients who have been shown to have an increased tendency to develop infection following surgery.

We found no statistically significant differences in the two groups with respect to delayed healing, sore throat and abscess formation following surgery. These results were inconsistent with previous studies [14].

Usually the administration of antimicrobials, mouthwashes and the maintenance of good oral hygiene have a great effect in preventing or treating such complications to a certain extent.

### Nerve injury

Previous studies have shown the incidence of damage to the lingual nerve following mandibular third molar surgery varied from 0% [18] to 23% [19] and that of the inferior alveolar nerve from 0.4% [6] to 8.4% [20]. The incidences of temporary nerve paraesthesia and permanent nerve dysfunction in our study are in keeping with these studies, irrespective of the surgeon's grade.

The OMFS residents reported higher incidence of lingual nerve paraesthesia than their specialist colleagues during the follow-up period. Permanent nerve dysfunction was considered to have occurred two years following surgery and our results show that the resident-treated group were four times more likely to develop this complication. Previous studies have shown that such an incidence may relate to the surgeon's experience, improper use of forceps and poor instrument handling [21].

The resident-group treated slightly higher numbers of patients whose impacted third molar teeth were considered to be close to the inferior dental nerve. This may explain the higher incidence of permanent inferior dental nerve injury in our resident-treated group. They were found to be seven times more likely to induce this complication when compared to the resident-group. The incidence of the permanent damage of the inferior dental and lingual nerves were found to be lower than the incidences reported by Bataineb [22] for both the senior and junior staff and quiet consistent with the results of Sisk et al. [6] for the specialists; while the residents had a lower incidence rate.

If the numbness persists by the end of the monitoring period (6 m-2 y), a further radiograph is required to assess the continuity of the mandibular canal (in case of the inferior dental nerve), and surgical exploration and decompression or repair of the nerve is considered; while, regarding the lingual nerve, surgical exploration is required to check the continuity of the nerve – if the nerve is not intact, a microsurgical repair is required [23].

### Dry socket

The OMFS resident-treated group were found to be three times more likely to develop alveolar osteitis. They, however, had a higher proportion of female patients (58.5%) who are more susceptible to this complication [24]. The incidence of dry socket also shows marked increase in smokers or patients taking oral contraceptives [24] following surgical removal of third molars. Previous studies had shown that females in general, and especially those taking oral contraceptives are more likely to get alveolar osteitis, which is thought may be due to the estrogenic effect on blood coagulation, which can lead to an early fibrinolysis of the blood clot in the extraction socket [25].

Other factors possibly involved include age, medical status, tooth position, surgical technique, duration of surgery and skills. These results are similarly consistent with other studies [6,14].

Unfortunately, there is no successful method of preventing dry socket, but the incidence of this unpleasant complication can be reduced by employing a number of prophylactic measures, that include: avoiding unnecessary trauma or excessive force during surgery, careful debriding of the socket from any loose fragments, adequate postoperative instructions and advice to avoid smoking for at least 24 hrs following surgery.

## Conclusion

The higher rate of postoperative complications in the residents group suggests that at least some of the complications might be related to surgical experience. This raises a number of important issues related to training. Ideally, third molar removal should only be carried out by experienced practitioners and not by occasional surgeons, however, surgeons are not created by divine right and need training to gain the requisite level of experience. This will unfortunately result in a higher level of complications even when residents are closely supervised. Patients have the right to know who will be performing their surgery and might be unhappy with the increased risk of being treated by a trainee.

Further work needs to compare specialists of training programmes with different years of experience, using large cross – sectional studies.
